# Attitude and Belief of Healthcare Professionals Towards Effective Obesity Care and Perception of Barriers; An Updated Systematic Review and Meta-analysis

**DOI:** 10.34172/aim.2023.78

**Published:** 2023-09-01

**Authors:** Mohammad E. Khamseh, Zahra Emami, Aida Iranpour, Reyhaneh Mahmoodian, Erfan Amouei, Adnan Tizmaghz, Yousef Moradi, Hamid R Baradaran

**Affiliations:** ^1^Endocrine Research Center, Institute of Endocrinology and Metabolism, Iran University of Medical Sciences, Tehran, Iran; ^2^Research Center for Prevention of Cardiovascular Disease, Institute of Endocrinology and Metabolism, Iran University of Medical Sciences, Tehran, Iran; ^3^Department of Internal Medicine, Alborz University of Medical Sciences, Karaj, Iran; ^4^Department of Internal Medicine, Iran University of Medical Sciences, Tehran, Iran; ^5^Department of Surgery, Iran University of Medical Sciences, Tehran, Iran; ^6^Department of Epidemiology and Biostatistics, School of Medicine, Kurdistan University of Medical Sciences, Sanandaj, Iran; ^7^Ageing Clinical & Experimental Research Team, Institute of Applied Health Sciences, University of Aberdeen, Scotland, UK

**Keywords:** Behavior, General practitioners, Obesity, Overweight, Physician, Primary health care

## Abstract

**Background:**

Obesity is a serious chronic disease that adversely affects health and quality of life. However, a significant percentage of people do not participate in or adhere to weight loss programs. Therefore, a multidisciplinary approach is needed to identify critical barriers to effective obesity management and to examine health practitioners’ attitudes and behaviors towards effective obesity treatment.

**Methods:**

This systematic review was conducted in accordance with PRISMA 2020. Eligible studies were identified through a systematic review of the literature using Medline, Scopus, Cochrane, Google Scholar, Web of Science, and Embase databases from January 1, 2011 to March 2, 2021.

**Results:**

A total of 57 articles were included. Data on 12663 physicians were extracted from a total of 35 quantitative articles. Some of the most commonly perceived attitude issues included "obesity has a huge impact on overall health", "obesity is a disease" and "HCPs are to blame". Health professionals were more inclined to believe in "using BMI to assess obesity," "advice to increase physical activity," and "diet/calorie reduction advice." The major obstacles to optimal treatment of obesity were "lack of motivation", "lack of time" and "lack of success".

**Conclusion:**

Although the majority of health care professionals consider obesity as a serious disease which has a large impact on overall health, counseling for lifestyle modification, pharmacologic or surgical intervention occur in almost half of the visits. Increasing the length of physician visits as well as tailoring appropriate training programs could improve health care for obesity.

## Introduction

 Obesity is a chronic and important disease with increasing prevalence. In 2016, more than 1.9 billion people over the age of 18 were overweight, of whom 650 million were obese. The global prevalence of obesity nearly tripled between 1975 and 2016.^1^ Additionally, only a minority of obese patients receive appropriate treatment, including lifestyle changes, pharmacological or surgical interventions.^2-5^ Much more attention must be paid to the critical role of medical staff in achieving optimal outcomes in managing obesity, despite well-known obstacles such as poor patient motivation, non-adherence to treatment, and short appointment times.^6^

 Previous studies have shown that health professionals, including family physicians and general practitioners, lack sufficient understanding and competence regarding obesity, which is a barrier to effective obesity care.^7^ Since there are insufficient and inconsistent limited reviews, we performed this systematic review in order to explore the attitude and behavior of health-care professionals and perception of barriers towards effective obesity care.

## Materials and Methods

**Figure 1:**
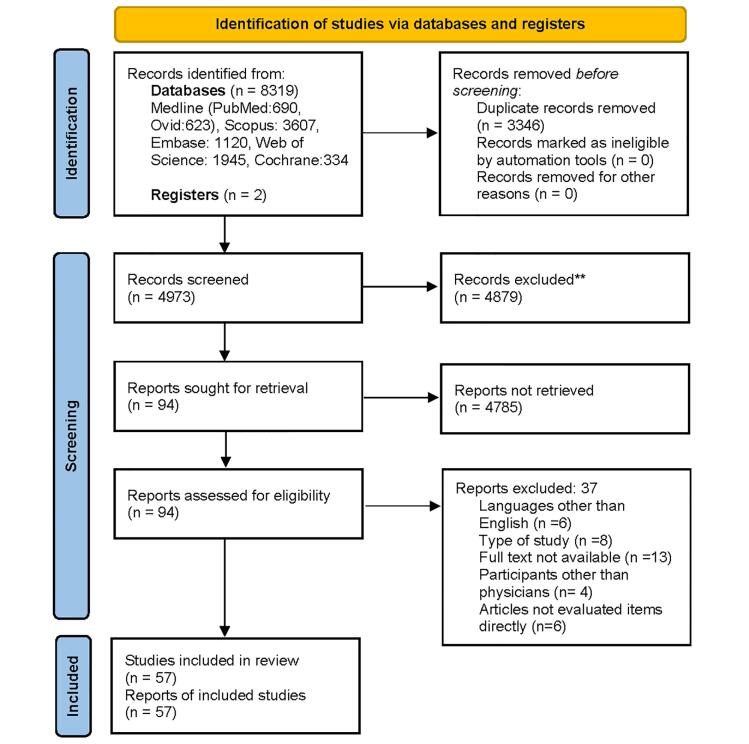
PRISMA 2020 Flow Diagram for Systematic Reviews with Database and Register Searches Only

 This systematic review was conducted in accordance withPreferred Reporting Items and checklist for Systematic Reviews and Meta-Analyses (PRISMA) 2020 (Figure 1) registered in PROSPERO (registration number: CRD42020148596) and was approved by Ethical committee of Iran University of Medical Sciences. (Ethical code: IR.IUMS.REC.1399.315)

 We searched Medline, Embase, Scopus, Cochrane Library, and Web of Science databases from January 1, 2011 to March 2, 2021 to identify relevant reports. Gray literature, including Google Scholar, was also explored. The search terms selected according to Medical Subject Heading (MESH) terminology included: “Obesity”, “overweight”, “belief”, “barrier”, “attitude”, “behavior”, “behavior”, “Physician”, “General Practitioner”, “health care provider”, “health care worker” using AND/OR in title and abstract.

 A preliminary database search using the Endnote program identified 8319 articles, including two articles identified through the gray literature sources (Google Scholar). After removing duplicates, 4973 records remained and were reviewed by two investigators. Of these, 4879 articles were excluded through title and abstract verification. The remaining 94 full-text articles were evaluated using the following inclusion criteria:

- Articles on adult obesity (excluding pediatrics)
- English-language articles
- Observational studies including case-control studies, cross-sectional studies and cohort studies
- Articles available in full text
- Articles which directly assess attitudes, beliefs and barriers to obesity care
- Articles addressing medical professionals including doctors (excluding articles about patients).

The reasons for excluding 37 records were: non-English language (n = 6), study type (n = 8), full text unavailable (n = 13), non-physician participants (n = 4), articles not relevant to aims of study (n = 6). Finally, we performed quality assessment of the remaining 57 articles using the JBI Critical Assessment Checklist for Cross-sectional Analysis Studies and scored on a 7-point scale.

###  Statistical Analysis

 The DerSimonian-Liard random-effects model was used to estimate the pooled prevalence using the Metaprop command in Stata 16. Cochrane Q and I^2^ tests were used to evaluate the heterogeneity and variance between studies. We used the funnel chart and its graph to assess publication bias. All two-step statistical tests were considered at α = 0.05.

## Results

 A total of 57 articles were included between 2011 and 2020. Of these, 12 articles were on barrier perceptions, half of which (6 articles) provided quantitative statistical data and the rest identified barriers qualitatively. Thirty-one articles were on attitude, 22 of which focused on quantitative information, and the remaining nine articles addressed qualitative information. The remaining 14 articles were on beliefs, with half of them being quantitative, and the rest qualitative. Different data collection methods were used such as Personal interviews, Telephone contacts, and Responses to surveys.

 A total of 35 quantitative articles were included (Table 1). The total number of participating physicians was 12 663. The largest study population was in the study of Steeves et al,^8^ which included 2022 individuals, and the smallest study population was in the study of McHale et al,^9^ which included 14 individuals.

**Table 1:** Main Characteristics of the Included Studies

**Chief Author**	**Year**	**Country**	**Participants**	**Response rate**	**Quality Assess (max=7)**	**Barriers**	**Attitude**	**Beliefs**
Steeves et al^8^	2014	USA	2022		5		HCPs have responsibility. Physician should be model or need a role model. Medical guidelines are effective.	
McHale et al^9^	2020	UK	14		7	Failure to able or success	A loss of 10% body weight would be beneficial to my overall health. Patients were aware of the health risks. Physician should be model or need to role model. Physicians’ role is to refer to professionals. Obesity is a disease.	
Glauser et al^10^	2015	USA	300		4	Failure to able or success Lack of referral options or educational resources Lack of interest Lack of training Stigma/feel uncomfortable or hard to talk	Obesity is a disease. Medical guidelines are effective.	Use BMI to assess obesity
Thapa et al^11^	2014	USA	52	92	3		Obesity is a disease. Physicians’ role is to refer to professionals. Medical guidelines are effective.	
Mkhatshwa et al^12^	2016	South Africa	48		4		Obesity is a disease. A loss of 10% body weight would be beneficial to my overall health. Patients can lose weight.	Counseling for lifestyle modification Assess obesity and document it
Srivastava et al^13^	2018	USA	61	41.8	3		Obesity is a disease. Obesity has a large impact on overall health and is associated with serious medical conditions.	
Rurik et al^14^	2013	Hungary	521	89	5		A loss of 10% body weight would be beneficial to my overall health. Physician should be model or need to role model. Obesity is a disease. Physicians’ role is to refer to professionals.	Counseling for increasing physical activity Follow treatment guidelines or provide educational materials Counseling for eating habits/reducing calories Use BMI to assess obesity Counseling for lifestyle modification
Dicker et al^15^	2020	Israel	169	21		Lack of interest Lack of motivation More important concerns to discuss Failure to able or success Stigma / feel uncomfortable or hard to talk Lack of physicians’ confidence	Obesity is a disease. A loss of 10% body weight would be beneficial to my overall health. HCPs have responsibility. Physicians’ role is to refer to professionals. Patients can lose weight.	
Epling et al^16^	2011	USA	75	36.5	5	Lack of time Lack of referral options or educational resources	Obesity has a large impact on overall health and is associated with serious medical conditions. Obesity is a disease. Educating patients is important. Physician should be model or need to role model. A loss of 10% body weight would be beneficial to my overall health. Patients were aware of the health risks. Patients can lose weight.	Refer to visiting a nutritionist or dietician
Huepenbecker et al^17^	2018	USA	134	42	5	Lack of training Lack of referral options or educational resources Lack of motivation	Educating patients is important. HCPs have responsibility. Medical guidelines are effective.	
Leiter et al^18^	2015	United Kingdom	335	42	3		Obesity has a large impact on overall health and is associated with serious medical conditions. Obesity is a disease. Medical guidelines are effective. A loss of 10% body weight would be beneficial to my overall health. Patients were aware of the health risks.	Refer to a nutritionist or dietician Follow treatment guidelines or provide educational materials Screen and assess related risks and disease Counseling for increasing physical activity Counseling for eating habits/reducing calories Prescription of weight loss medication Counseling for weight loss surgery
Sebiany et al^19^	2013	Saudi Arabia	130	87	5	Lack of training Lack of compliance Lack of referral options or educational resources Lack of time Failure to able or success Lack of physicians’ confidence	Obesity has a large impact on overall health and is associated with serious medical conditions. Obesity is a disease.	Follow treatment guidelines or provide educational materials
Simon et al^20^	2018	USA	111	26	4	Lack of time Lack of training Stigma / feel uncomfortable or hard to talk		Assess obesity and document it Counseling for eating habits/reducing calories Counseling for increasing physical activity Counseling for lifestyle modification Follow treatment guidelines or provide educational materials Counseling for weight loss surgery Refer to a nutritionist or dietician Prescription of weight loss medication
Attalin et al^21^	2011	France	203	80	4	Lack of compliance Lack of referral options or educational resources Lack of time Failure to able or success Lack of training	A loss of 10% body weight would be beneficial to my overall health.	Counseling for increasing physical activity Counseling for eating habits/reducing calories Behavior therapy or psychotherapy Refer to visiting a nutritionist or dietician
Alshammari et al^22^	2014	Saudi Arabia	130	87.2	5		Obesity is a disease. A loss of 10% body weight would be beneficial to my overall health. Physician should be model or need to role model. Physicians’ role is to refer to professionals.	Counseling for increasing physical activity Counseling for eating habits/reducing calories Use BMI to assess obesity Screen and assess related risks and disease follow treatment guidelines or provide educational materials Counseling for WL surgery Refer to visiting a nutritionist or dietician Behavior therapy or psychotherapy Prescription of WL medication
Petrin et al^23^	2017	USA	1501	77	3		HCPs have responsibility. Medical guidelines are effective.	Screen and assess related risks and disease Counseling for increasing physical activity Start discussing or Motivational interviewing Counseling for eating habits/reducing calories
Sharma et al^24^	2019	Canada	395	34	3	Lack of interest Lack of time Lack of motivation More important concerns to discuss	Obesity is a disease. HCPs have responsibility. Obesity has a large impact on overall health and is associated with serious medical conditions. Patients were aware of the health risks. Medical guidelines are effective.	Counseling for eating habits/reducing calories Counseling for increasing physical activity Follow treatment guidelines or provide educational materials Screen and assess related risks and disease Behavior therapy or psychotherapy Refer to visiting a nutritionist or dietician Prescription of weight loss medication Counseling for weight loss surgery
Tsai et al^25^	2017	USA	272		2			
Kaplan et al^26^	2017	USA	606	20.4	4	Lack of time Lack of motivation More important concerns to discuss Lack of interest Stigma/feel uncomfortable or hard to talking	A loss of 10% body weight would be beneficial to my overall health. Obesity is a disease. HCPs have responsibility. Obesity has a large impact on overall health and is associated with serious medical conditions.	Start discussing or Motivational interviewing Counseling for eating habits/reducing calories Counseling for increasing physical activity Assess obesity and document it Counseling for lifestyle modification Refer to visiting a nutritionist or dietician Prescription of weight loss medication Counseling for weight loss surgery
Hite et al^27^	2018	USA	206	74	4	HCPs misdiagnosis Lack of time lack of physicians’ confidence Lack of compliance Lack of referral options or educational resources Stigma / feel uncomfortable or hard to talking	Patients were aware of the health risks.	Assess obesity and document it Use BMI to assess obesity Start discussing or Motivational interviewing Screen and assess related risks and disease
Aleem et al^28^	2015	USA	51	91	5	Lack of time Lack of motivation Stigma / feel uncomfortable or hard to talk More important concerns to discuss Lack of training		Counseling for increasing physical activity Assess obesity and document it Start discussing or Motivational interviewing Refer to visiting a nutritionist or dietician
Wong et al^29^	2018	Australia	204	81.6	2			Counseling for eating habits/reducing calories Counseling for increasing physical activity Refer to visiting a nutritionist or dietician Follow treatment guidelines or provide educational materials Prescription of weight loss medication Counseling for weight loss surgery
Turner et al^30^	2018	USA	1506	64.5	5		Medical guidelines are effective.	
Alzouebi et al^31^	2012	United Kingdom	100	73	0	Lack of training		
Aucott et al^32^	2011	United Kingdom	194	51	3		Obesity is a disease.	Assess obesity and document it
Granara et al^33^	2017	USA	94	11	4	Failure to able or success Lack of motivation Lack of referral options or educational resources Lack of training Lack of time		
Laidlaw et al^34^	2019	Scotland	107	18.5	3	Lack of time Lack of motivation Lack of referral options or educational resources More important concerns to discuss Lack of training Lack of physicians’ confidence	A loss of 10% body weight would be beneficial to my overall health. HCPs have responsibility. Physician should be model or need to role model. Obesity is a disease. Physicians’ role is to refer to professionals.	Use BMI to assess obesity
Look et al^35^	2019	USA	606		5			Start discussing or Motivational interviewing
Mudi et al^36^	2019	Bangladesh	300		2			
Saedon et al^37^	2015	Brunei	77	85.6	6		Patients can lose weight. Physicians’ role is to refer to professionals. Patients were aware of the health risks.	
Sikorski et al^38^	2013	Germany	682		6			
Smith et al^39^	2015	USA	219	62	5			
Bąk-Sosnowska et al^40^	2015	Poland	250		3			Counseling for eating habits/reducing calories Start discussing or motivational interviewing Counseling for increasing physical activity Counseling for lifestyle modification Screen and assess related risks and disease
Goranova-Spasova et al^41^	2020	Bulgaria	154		5	Lack of physicians’ confidence More important concerns to discuss Lack of time Lack of motivation	Physicians’ role is to refer to professionals. HCPs have responsibility.	

BMI, body mass index; HCPs, health care providers; WL, weight loss.

###  Attitudes

**Figure 2:**
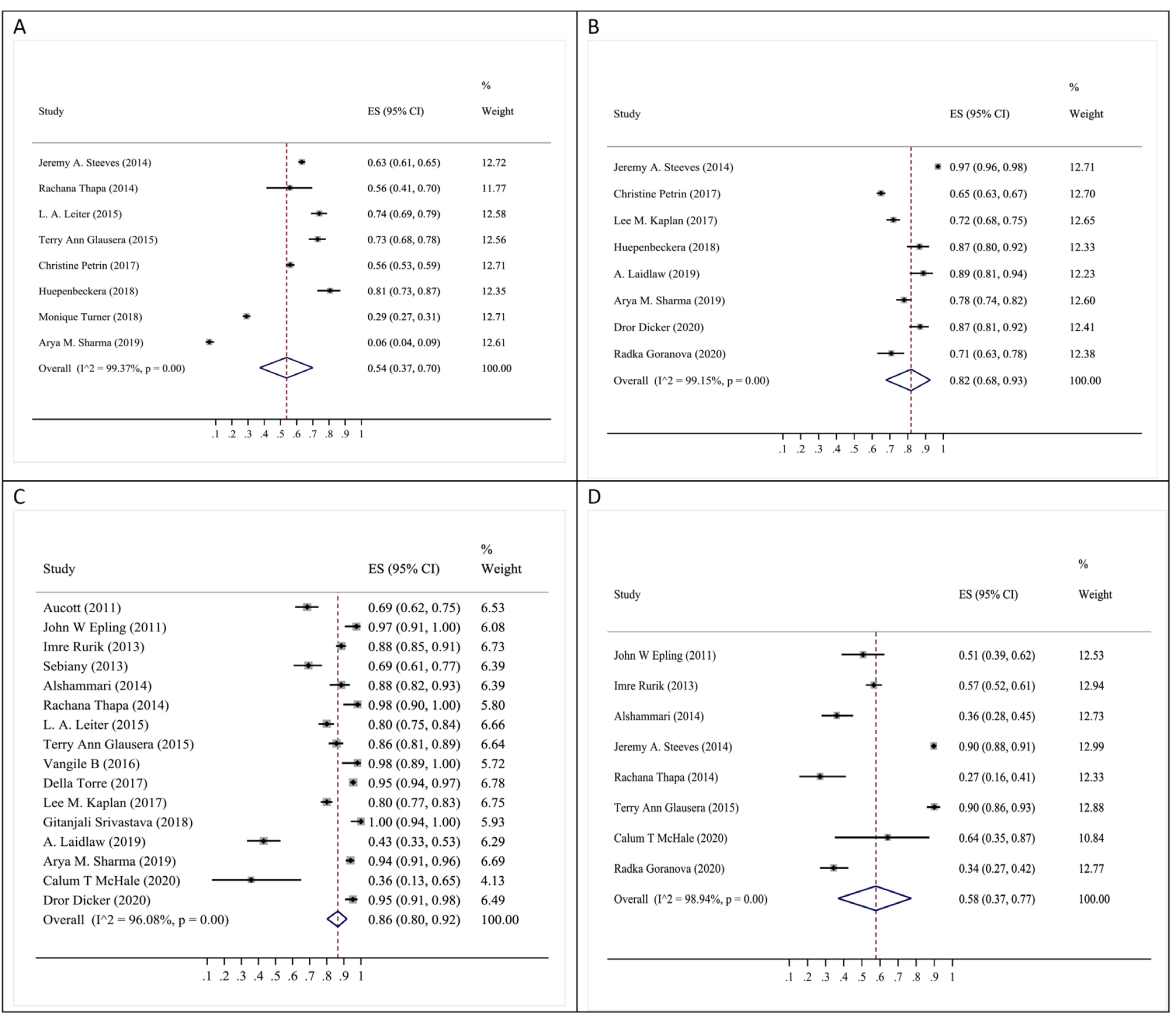
The Pooled Prevalence of Attitude Scales in Healthcare Professionals Towards Effective Obesity Care (A: Medical guidelines is effective, B: HCPs have responsibility, C: Obesity is a disease, and D: HCPs management will success and feel confident)

 Some of the most commonly identified attitudes between health care providers (HCPs) included:

 “Obesity has a significant impact on overall health and is associated with serious illness” (87%; 95% CI: 71‒98%), “Obesity is a disease” (86%; 95% CI: 80‒98%), and “HCPs are responsible for obesity in their communities” (82%; 95% CI: 68‒93%) (Figure 2 and Table 2; Supplementary file 1).

**Table 2:** The Quantitative Results and Pooled Prevalence of Scales Based on Meta-analysis Results

**Status**	**Pooled Prevalence (%)**	**95 % CI (Lower and Upper)**
Barriers		
Lack of motivation	53 %	39– 67 %
Lack of time	46 %	32 – 59 %
Lack of referral options or resources	38 %	21 – 56 %
Lack of training	37 %	16 – 60 %
More important concern to discuss	38 %	30 – 47 %
Lack of physician’s confidence	19 %	6 – 38 %
Failure to able or success	42 %	27 – 59 %
Stigma/feel uncomfortable or hard to talking	14 %	8 – 21 %
Attitude		
Obesity has a large impact on overall health and associated with serious medical conditions	87 %	71 – 98 %
Obesity is a disease	86 %	80 – 98 %
HCPs have responsibility	82 %	68 – 93 %
A loss of 10% body weight would be beneficial to my overall health	78 %	61 – 91 %
Physician should be model or need to role model	76 %	64 – 86 %
HCPs management will success and feel confident	58 %	37 – 77 %
Medical guidelines are effective	54 %	37 – 70 %
Physician’s role is to refer to professionals	53 %	30 – 76 %
Patients were aware of the health risks	39 %	23 – 56 %
Beliefs		
Using BMI to assess obesity	78 %	67 – 87 %
Counseling for increasing physical activity	68 %	53 – 81 %
Counseling for eating habits/reducing calories	64 %	51 – 76 %
Counseling or lifestyle modification	61 %	34 – 85 %
Discussing or Motivational interviewing	57 %	43 – 71 %
Follow treatment guidelines or provide educational materials	53 %	29 – 76 %
Assess obesity and document it	55 %	26 – 82 %
Screen and assess related risks and disease	49 %	18 – 80 %
Refer to visiting a nutritionist or dietician	25 %	13 – 40 %
Prescription WL medication	10 %	6 – 13 %
Counseling for WL surgery	13 %	4 – 24 %

“10% weight loss helps overall health” (78%; 95% CI: 61‒91%), “Physicians should be or need to be role models” (76%; 95% CI: 64‒86%), “HCP leadership in obesity management is prosperous and secure” (58%; 95% CI: 37‒70%) (Figure 2 and Table 2; Supplementary file 1).

 Funnel plots to assess publication bias are reported in Supplementary file 1.

###  Beliefs

**Figure 3:**
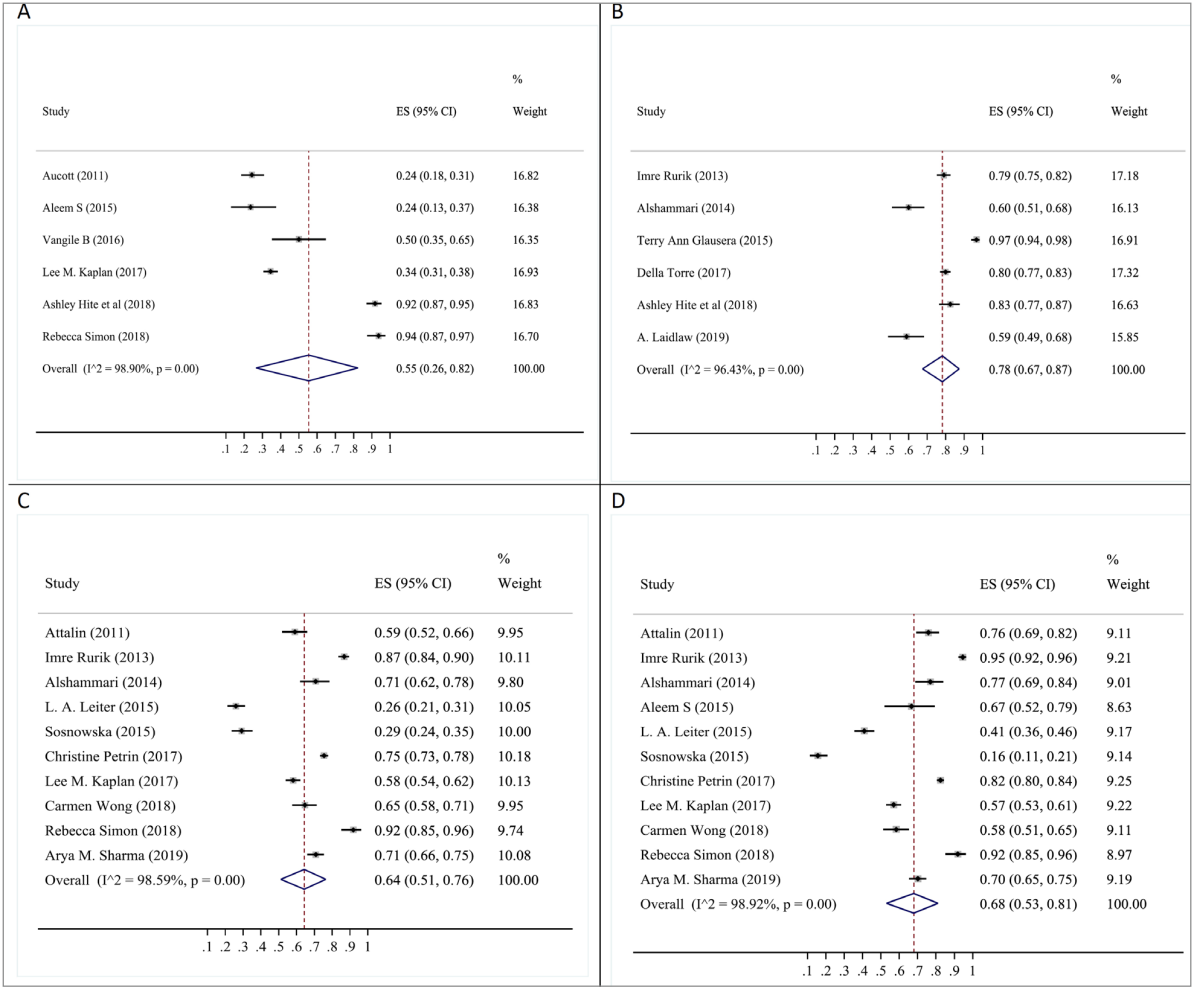
The Pooled Prevalence of Belief Scales in Healthcare Professionals Towards Effective Obesity Care (A: Assess obesity and document it, B: Use BMI to assess obesity, C: counselling for eating habits/reducing calories, and D: counselling for increasing physical activity)

 When considering quantitative data, the three most common beliefs were “BMI is used to assess obesity” (78%; 95% CI: 67‒87%), “Counseling to increase physical activity” (68%; 95% CI: 53–81%) and “Nutrition and calorie restricting advice” (64%; 95% CI: 51‒76%). Other common beliefs were related to «counseling to change lifestyle « (61%; 95% CI: 34‒85%), “Negotiation interview or Motivational interview” (57%; 95% CI: 43‒71%), “Follow treatment guidelines or provide educational materials” (53%; 95% CI: 29‒76%), «Assessment and documentation of obesity» (55%; 95% CI: 26‒82%) and “Screening and Assessment of associated risks and conditions” (49%; 95% CI: 18‒80%) (Figure 3 and Table 2; Supplementary file 1). Funnel plots to assess publication bias are reported in Supplementary file 1.

###  Barriers

**Figure 4:**
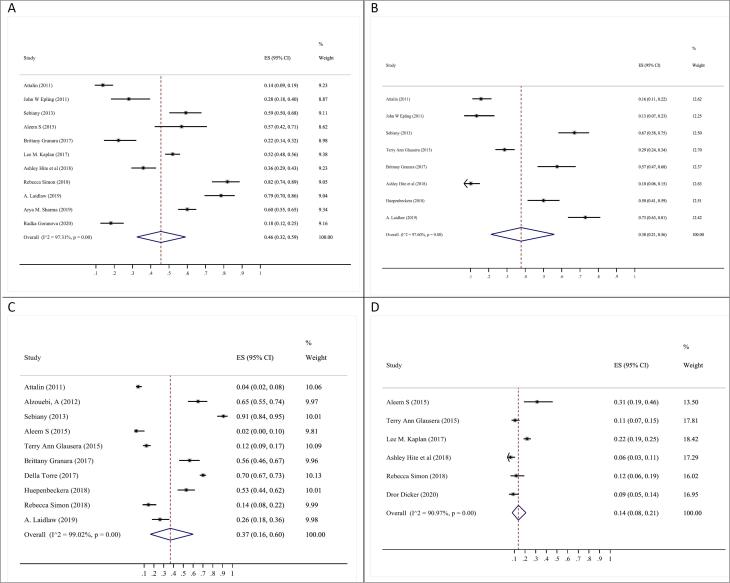
The Pooled Prevalence of Barriers Scales in Healthcare Professionals Towards Effective Obesity Care (A: Lack of time, B: Lack of referral options or educational resources, C: Lack of training, and D: Stigma / feel uncomfortable or hard to talking)

 The three biggest barriers to optimal obesity care were ‘lack of motivation’ (53%; 95% CI: 39‒67%), “I don’t have time” (46%; 95% CI: 32‒59%) and “Failure or inability to achieve the desired outcome” (42%; 95% CI: 27‒59%) (Figure 4, Table 2). Other barriers were “lack of education” (37%; 95% CI: 16-60%), “lack of referral facilities or resources” (38%; 95% CI: 21‒56%) and “having more important issues to discuss” (38%; 95% CI: 30‒47%) (Figure 4 and Table 2; Supplementary file 1).

 Funnel plots to assess publication bias are reported in the Supplementary File. All of the qualitative studies mentioned that “lack of clearly specified practical guidelines and treatment options” are the main barriers. In addition, “limited understanding about obesity care, knowledge and skills” was the second major barrier.

## Discussion

###  Belief and Attitude 

 Obesity-related beliefs and attitudes refer to conscious perceptions, interpretations, and thoughts or feelings of obesity that lead to understanding and learning. This may motivate HCPs to provide the highest quality care.^11^ The majority of physicians consider obesity as a disease; however, obesity is not just about increased BMI. It is associated with changes in anthropometrics, metabolic function, and gait parameters. Glauser et al reported that the majority of respondents used body mass index for obesity screening.^10^ Considering the limitations of BMI and the importance of central obesity, body fat distribution which is closely linked to metabolic complications should be taken into account.^8^ Waist circumference and waist-to-hip ratio could reliably distinguish central obesity from lower body and general obesity.^42^

 Approaches to assessing obesity are multifaceted. Approximately 50% of physicians documented obesity as a problem, with attending physicians documenting obesity more frequently than residents (64% vs. 43%). On the other hand, normal-weight physicians reported obesity more frequently than overweight physicians (58% vs. 41%). Furthermore, doctors may not recognize obesity as a serious problem unless the patient’s BMI exceeds 35 kg/m^2^. Educational programs for health professionals are therefore needed to detect obesity early and increase the ratio of documentation. This could be a first step towards improving obesity management.^11^

 Regarding the physicians’ perceptions of obesity, most HCPs and general practitioners (GPs) view obesity as a chronic disease. They also believe that obesity has a significant impact on overall health and is linked to serious medical conditions.^11-13^

 In addition, the majority of health care professionals accept their responsibility to improve the situation and reducing the prevalence of this chronic disease. Moreover, they believe that they need to maintain a healthy weight in order to serve as a role model for their patients.^14,15^ However, a study by Tiexeira et al showed that although GPs believe that counseling obese patients about health risks is part of their job, their perception about their role in treating obesity is negative. The majority of doctors believe that they make no difference in getting their patients to make long-term lifestyle modification.^7^ Patient-centered communication, motivational interviewing, problem-solving, and action planning should be integrated into residency curricula and local continuing education programs.

 Regarding interviews, Kirk et al showed that within the current healthcare system, HCPs are unable to provide the support obese people really need. They identified tensions within three themes expressed by groups of participants: “*blame as a devastating relation of power, tensions in obesity management and prevention, and the prevailing medical management discourse”.*^43^ These are reflected tensions and varying discourses surrounding obesity, namely obesity as a personal issue, obesity as a social construction, and obesity as a complex health condition. The study by Heintze et al showed that while almost all of the HCPs felt responsible for providing obesity-related care, they felt they are incompetent.^44^ Several factors that are related to physician characteristics, such as physician BMI and specialty, may explain this issue. For example, normal-weight physicians reported more confidence in counseling patients compared to their overweight peers. Additionally, obese patients also reported more trust in receiving weight loss advice from normal-weight physicians.^44^ Similar beliefs can be applied to many specialists. Pediatricians and obstetricians felt limited impact on treatment efficacy compared to other disciplines, and this finding may be considered in continuing medical education programs.

 One of the important issues about attitude toward obesity is patient awareness. Physicians believe that nearly half of patients neither have the necessary knowledge about obesity and its harms, nor the ability to lose weight. Hence, they will lose the battle. That is why the majority of HCPs and GPs believe that patient education improves this situation.^16,17^ On the other hand, about half of the HCPs and GPs mentioned the need for updating the existing guidelines.^10,17^ On the other hand, the perception of physicians about treatment modalities is quite diverse. While about 54% of participants believe that current guidelines are effective,^8,10,17,18^ implementation of guidelines into daily practice is still far from the desired standards. Only half of the participants (53%) follow treatment guidelines or provide educational materials.^14,19,20^ As a rule, the combination of dietary modification and exercise is the most effective behavioral modality for the treatment of obesity. In this regard, over 60% of physicians provide advice to their patient to improve physical activity^20-23^ and make dietary modifications.^20,23,24^ Comparing different treatment modalities, many physicians expected larger weight loss with pharmacotherapy and surgery. The majority of primary care providers, endocrinologists, and cardiologists expected less weight loss with gastric bypass surgery while bariatric surgeons had a more reasonable expectation; a finding that could be explained by different educational and practical backgrounds.^8,10^ The study by Tsai et al revealed that physicians believe medication and surgery are less effective in comparison to lifestyle modification alone; thus, physicians who better understand the biology might be more open to using ‘biologic’ tools (medications and surgery). Perhaps, lack of valid and clear guidelines, as well as lack of proper awareness of the side effects and benefits of various interventions can play a role in this issue.^25^ Bleich et al confirm this fact that primary care physicians overwhelmingly supported additional training to improve nutrition and exercise counselling, optimal care related to bariatric surgery patients, as well as motivational interviewing.^45^ Primary care providers in this study also identified nutritionists/dietitians as the most qualified providers for obesity care.

###  Barriers to Optimal Obesity Management 

####  Patient Factors

####  Lack of Motivation for Weight Loss and Lack of Compliance for Maintaining Long-term Lifestyle Changes

 Obesity should be considered and managed as a medical condition that is progressive, chronic, and relapsing.^46^ In this study, nearly 54% of physicians believed that patients with obesity are not motivated enough to lose weight. In addition, almost 50% of healthcare professionals believe that people with obesity are non-compliant with long-term lifestyle changes. In a study by Kim et al in 2020, about 80% of people with obesity had experienced at least one serious weight-loss effort in the past.^47^ Interestingly, only 12% of these people had successfully lost near 10% of their body weight, half of whom kept it off for at least one year.^47^ The relatively high failure rate can be partially attributed to lack of long-term adherence to the obesity treatment. Therefore, despite successful weight loss after bariatric surgery, weight regain may occur, especially if they are less motivated and fail to adhere to long-term lifestyle changes. Thus, as with other chronic diseases, patients with obesity require to understand that obesity is a serious, chronic, relapsing recurring disease, and maintaining adherence to lifelong lifestyle changes is crucial for the long-term weight loss maintenance.

####  Lack of Referral Options or Resources (Misbelief and Misinformation Around Obesity Treatment) and Lack of Training

 People with obesity have different attitudes and beliefs about obesity and its management. Interestingly, a great number of people with obesity prefer to seek advice from informal sources of information such as friends, family, and websites, rather than a qualified healthcare professional.^47-49^ According to this review, about 40% of healthcare professionals believe that patients with obesity would rather seek out medical advice themselves instead of visiting a licensed professional.

####  Stigma

 Stigma is a common problem affecting the disabled community. Furthermore, it can lead to significant financial disadvantage; opportunities are denied and self-esteem is compromised. Many people with obesity feel that they are labelled as unmotivated, lazy, and uncooperative. According to this review, approximately 15% of health professionals reported having a negative attitude towards obesity. The negative attitude of health professionals towards obesity could be a potential barrier to optimal obesity care. Therefore, the medical profession should be more understanding, be compassionate with the patients and help them to lose weight. In addition, the negative attitudes of other members of the health care team and of course, the society towards the population with obesity need to be improved.^50^

###  Physician Factors

####  Lack of Time During General Practice Appointments 

 One of the most common obstacles to obesity management is limited length of the visits.^51-54^ Therefore, most physicians do not consistently address the issue of overweight or obesity directly with their patients.^26,52,55^ According to this review, about 46% of healthcare professionals indicated that they do not talk about weight management with their patients because they do not have enough time in their appointments.

####  HCPs Misdiagnosis and Lack of a Formal Diagnosis of Obesity

 Sometimes, it is difficult for healthcare professionals to diagnose obesity by means of visual inspection alone and therefore, obesity may not be addressed during a patient care visit. According to this review, the accuracy of visual assessment of BMI was 45%. As a result, in order to appropriately diagnose and manage obesity, the BMI of a patient must be consistently calculated by all practitioners.^27,56,57^

####  Lack of Training and Obesity Counseling Competence (Lack of Expertise)

 Although diet, nutrition, exercise, behavior therapy, and medication are among topics covered in obesity education for healthcare professionals, about 40% cited that they encountervarious challenges to aligning their clinical practice with current obesity management guidelines. This leads to the conclusion that most medical school curricula do not encompass sufficient obesity education and thus, medical schools must adequately address obesity education in their curricula, including adequate nutrition and obesity medication education.^47,58,59^

####  More Important Concern to Discuss

 Discussions about the possible impacts of obesity on general health are a potentially disturbing and humiliating topic.^51^ In this study, almost 38% of the clinicians believed that there are moreimportant clinical issues to discuss during their appointment rather than weight management. The majority of healthcare professionals (62%) indicated that they are very willing to discuss weight control issues with patients, but report that there are obstacles to starting these conversations.

####  Lack of Physician’s Confidence

 Physicians often report a lack of confidence in managing obesity. Among healthcare providers, physicians with a normal BMI are more confident in their capacity to provide patients with obesity diet, exercise, and counseling.^45^ About 22% of the physicians in this review expressed lack of confidence in their ability to manage obesity.

## Conclusion

 Evaluation of attitudes, beliefs and barriers toward effective obesity care revealed that although most of the physicians consider obesity as a serious disease which has a large impact on health, counseling for lifestyle modification, pharmacologic or surgical intervention occur in almost half of the visits. Therefore, tailoring appropriate training programs is needed in order to improve the attitude and perception of health care professionals about optimal obesity care.

## Supplementary Files

Supplementary file 1 contains Figures S1-S3.
