# Supplementary material for: Attitude and Belief of Healthcare Professionals Towards Effective Obesity Care and Perception of Barriers; An Updated Systematic Review and Meta-analysis
Source: Arch Iran Med. 2023 Sep 1;26(9):529–41. doi: 10.34172/aim.2023.78 (PMC10862058; doi:10.34172/aim.2023.78)
Supplement: Supplementary file 1 — contains Figures S1-S3. [file aim-26-529-s001.pdf]

Arch Iran Med. September 2023;26(9):529-S1.

10.34172/aim.2023.78

Supplementary file 1

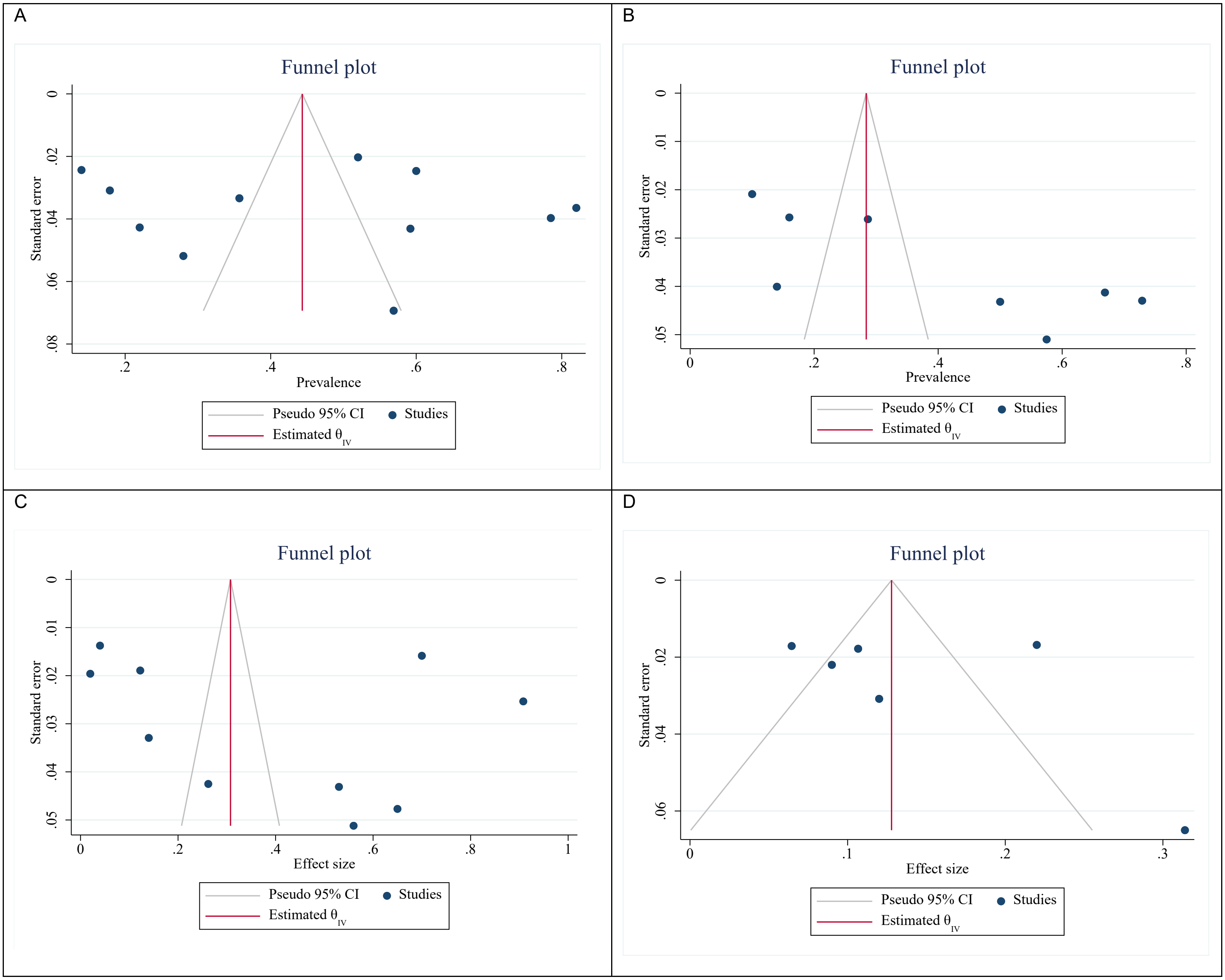

Figure S1. The funnel plot of barriers scales in health-care professionals towards effective obesity care (**A:** Lack of time, B: Lack of referral options or educational resources, **C:** Lack of training, and **D:** Stigma / feel uncomfortable or hard to talking)

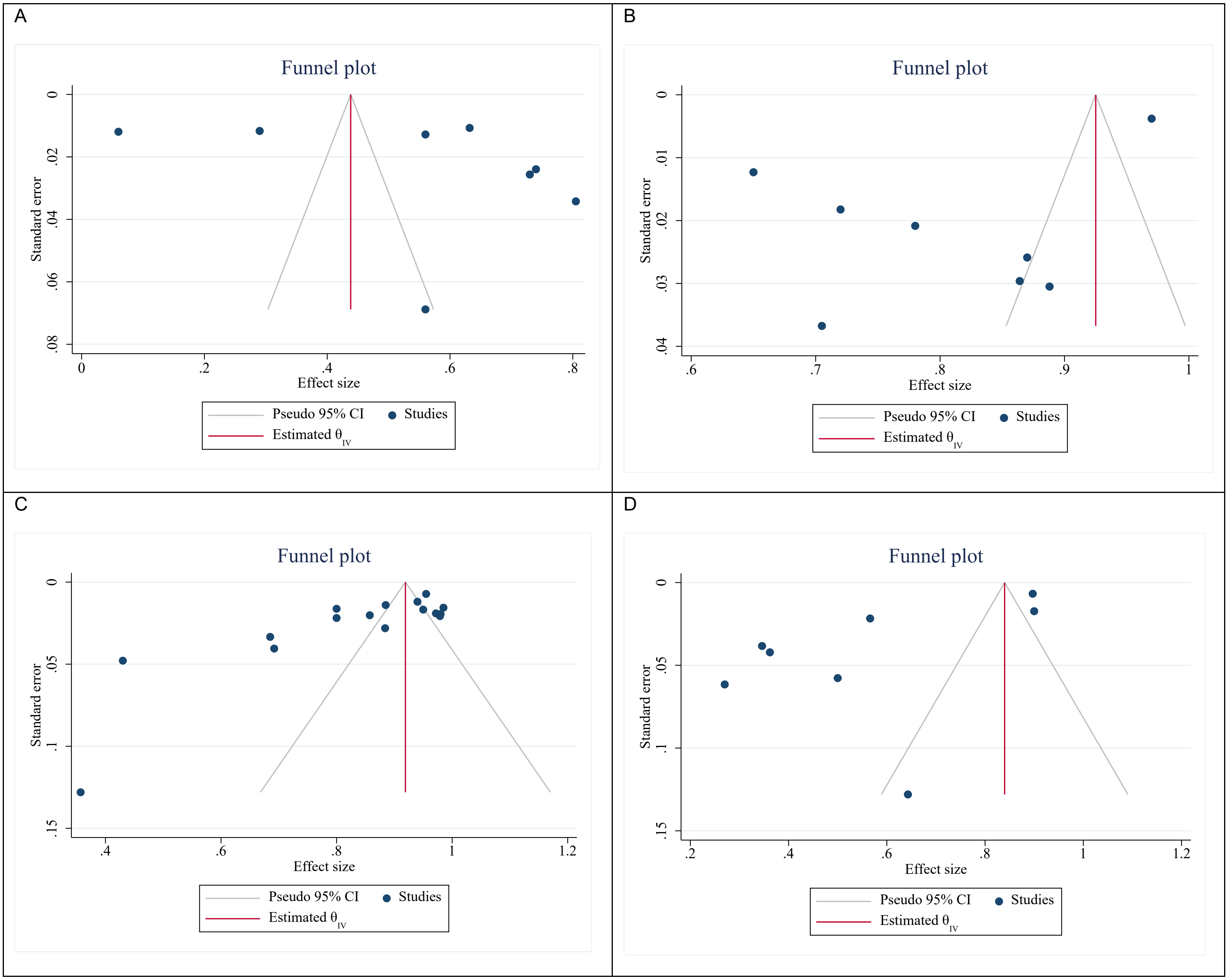

Figure S2. The funnel plot of Attitude scales in health-care professionals towards effective obesity care (**A:** Medical guidelines is effective, **B:** HCPs have responsibility, **C:** Obesity is a disease, and **D:** HCPs management will success and feel confident)

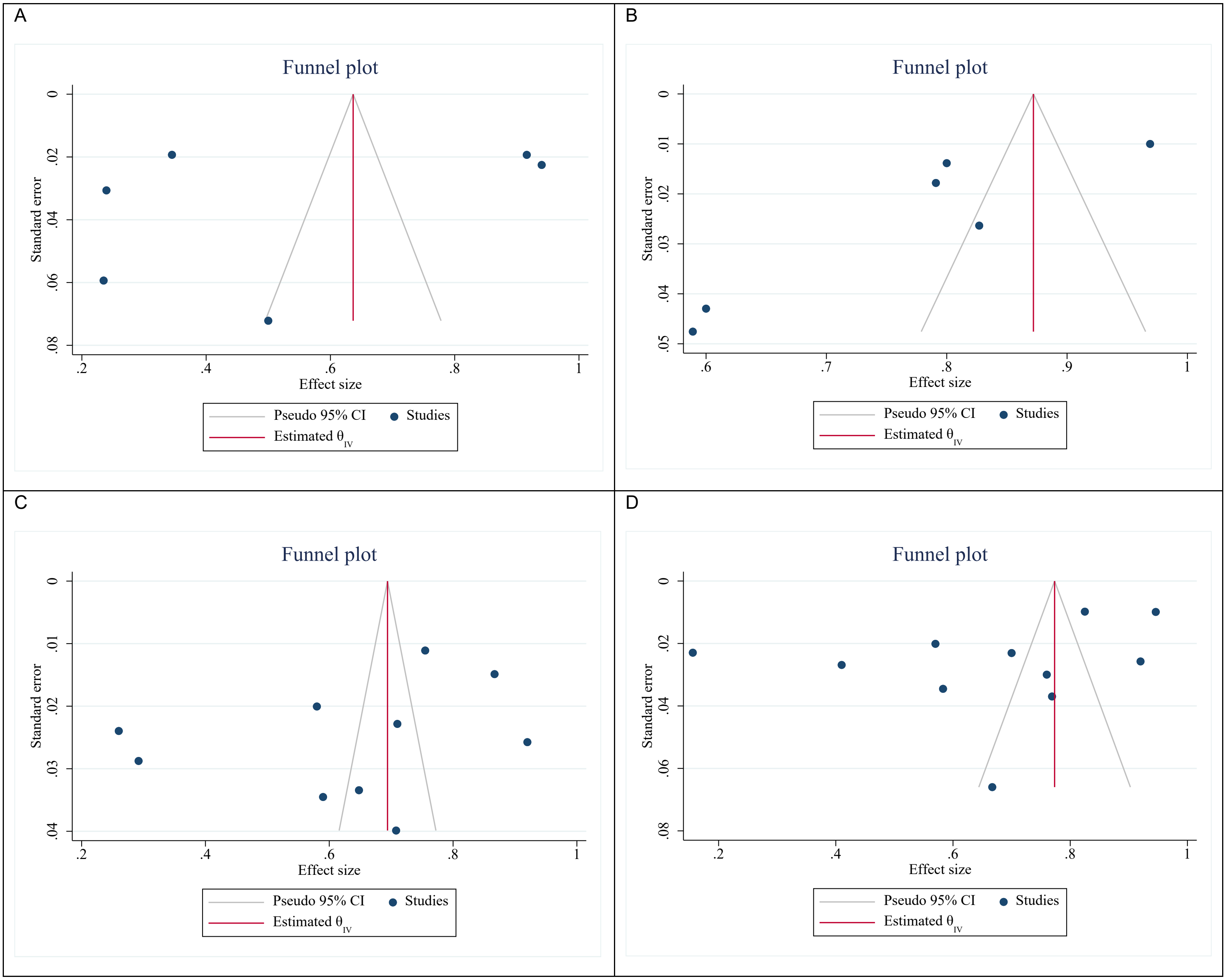

Figure S3. The funnel plot of Belief scales in health-care professionals towards effective obesity care (**A:** Assess obesity and document it, **B:** Use BMI to assess obesity, **C:** counselling for eating habits/reducing calories, and **D:** counselling for increasing physical activity)
